# Primary healthcare needs and barriers to care among Calgary’s homeless populations

**DOI:** 10.1186/s12875-015-0361-3

**Published:** 2015-10-13

**Authors:** David J. T. Campbell, Braden G. O’Neill, Katherine Gibson, Wilfreda E. Thurston

**Affiliations:** Departments of Medicine and Community Health Sciences, Cumming School of Medicine, University of Calgary, Room G236 Health Sciences Centre, 3330 Hospital Dr NW, Calgary, AB T2N 1 N4 Canada; Department of Family and Community Medicine, University of Toronto, Toronto, ON Canada; 1 Canadian Field Hospital, Canadian Armed Forces, Petawawa, ON Canada; Department of Community Health Sciences, Cumming School of Medicine, University of Calgary, Calgary, AB Canada

**Keywords:** Homeless, Poverty, Qualitative research, Needs assessment

## Abstract

**Background:**

Despite Canada’s universal healthcare system, significant barriers impede individuals experiencing homelessness from accessing health services. Furthermore, there is a paucity in the qualitative literature describing how Canadians experiencing homelessness access health care services. Our objective was to qualitatively explore perceived healthcare needs and barriers among individuals experiencing homelessness in one large Canadian city – Calgary, Alberta.

**Methods:**

We conducted a qualitative descriptive study that included open-ended interviews and focus groups with a variety of stakeholders who are involved in healthcare among Calgary’s homeless populations. These included individuals experiencing homelessness (*n* = 11) as well as employees from several healthcare service providers for those experiencing homelessness (*n* = 11). Transcripts from these interviews were thematically analyzed by two analysts.

**Results:**

Stakeholder interviews yielded several pervasive themes surrounding the health care needs of the homeless and barriers to accessing care. Some of the primary health care needs which were identified included mental health, addictions, and allied health as well as care that addresses the social determinants of health. Notably, it was difficult for many stakeholders to pinpoint specific health care priorities, as they identified that the health care needs among Calgary’s homeless populations are diverse and complex, often even describing the needs as overwhelming. Types of barriers to primary care that were identified by stakeholders included: emotional, educational, geographical, financial and structural barriers, as well as discrimination.

**Conclusions:**

Our findings highlight the diverse primary health care needs of Calgary’s homeless populations. Despite the fact that Canada has a universal publicly funded health care system, individuals experiencing homelessness face significant barriers in accessing primary care.

## Background

In 2012, the Calgary Homeless Foundation (CHF) determined that there were 3190 people experiencing homelessness in the City of Calgary [1]. Their 10 year plan to end homelessness states that for many individuals, poor health status and the lack of access to regular and reliable primary and specialized health services are significant barriers to accessing regular employment and stable housing situations [2]. There are several organizations and stakeholders providing services for Calgary’s homeless population. Services are decentralized, although the CHF encourages collaboration and coordination. It is unclear what the actual healthcare needs and barriers to accessing existing services are for individuals experiencing homelessness in Calgary, and what additional services might be required to support individuals who are currently homeless towards gaining housing, employment, and health.

While the health of individuals experiencing homelessness is determined by a large number of factors beyond healthcare, the focus of this study is on the role of primary healthcare. Primary healthcare is defined as “essential healthcare, based on practical, scientifically sound and socially acceptable methods and technology, made universally available to individuals and families in the community through their full participation and at a cost that the community and country can afford to maintain at every stage of their development in the spirit of self-reliance and self-determination” [3]. Another widely used definition describes it as an individual’s first point of contact with the healthcare system [4].

There are several homeless shelters in Calgary that provide emergency and transitional shelter to over 90 % of the city’s homeless [1]. The largest of these shelters is the Calgary Drop-in and Rehab Centre (known locally as the DI). It is Canada’s largest homeless shelter, housing over 1100 individuals experiencing homelessness on a nightly basis [5]. In 2006, the DI commissioned a study looking at where their clients accessed primary care services [6]. The study reported that those experiencing homelessness perceived their health to be poorer than the general population. The results showed that the vast majority of DI clients accessed primary care services on a walk-in basis at inner-city clinics and health centres. Since that report little work has been done, however, to better understand the health needs of Calgary’s ever-growing homeless population.

Dunlop, Coyte and McIsaac have shown that despite Canada’s provision of universal healthcare, socioeconomic status still plays a predictable role in the use of physician services [7]. Over the past several years, there have been a number of high quality quantitative studies documenting a lack of access to health services and poorer health outcomes among Canadians experiencing homelessness [8–11]. Furthermore, there is a robust body of evidence on homelessness and access to healthcare from comparable countries such as the United Kingdom [12–14] and the United States [15–17].

One qualitative study of Canadian Aboriginal women experiencing homelessness was recently published in a Canadian compilation [18]. There have also been relevant qualitative studies from Ontario published which documented the perceived healthcare needs of individuals experiencing homelessness [19]. However, there remains a relatively unexplored anecdotal need for primary care for inner-city homeless populations, as well as a lack of understanding of the specific barriers limit this population’s ability to access adequate health services and there is a need for further qualitative exploration of these issues.

## Methods

### Study objectives

This study had two aims. First, it aimed to enhance knowledge about perceived primary healthcare needs among urban homeless populations in Calgary. Second, it aimed to explore what barriers currently exist to meeting these needs.

### Study design

We conducted a qualitative descriptive study, as described by Sandelowski [20]. Qualitative description seeks to elicit and capture perspectives from a variety of stakeholders through qualitative methods such as interviews and focus groups. The results are then presented in a descriptive fashion. We conducted this study as a needs assessment, which is the systematic use of research tools to assess the gap between desired and existing services and/or their outcomes [21].

In order to obtain different perspectives on barriers to accessing primary care, a variety of stakeholder groups were represented among those included in this study, including individuals experiencing homelessness. Because the homeless population in Calgary is a diverse group comprised of varying ages and circumstances, careful consideration was taken to ensure that barriers to primary care access among different subpopulations were explored. Because another study was underway at a shelter for women fleeing abuse, that particular sub-population was not included in this study. Relevant stakeholder groups included staff and clients from the DI and five other organizations providing services to the target population. Each of these agencies served a different sub-population, and by including research participants from each, we were able to explore the perspectives of people in a wide range of health states and elicit a more comprehensive set of barriers to primary healthcare.

### Data collection

Data was collected using qualitative methods including face-to-face semi-structured individual interviews and focus groups. This approach was chosen because it is considered most appropriate for exploring and interpreting responses from multiple stakeholders [22, 23]. Key informants from relevant organizations were interviewed and were asked for their cooperation in recruiting client informants. Purposeful sampling [24] was used to recruit participants who could speak to issues around access to primary care and who also met the following criteria:

1. had been homeless for at least one week once in the last six months;
2. were 18 years of age or older;
3. could speak and understand English; and
4. had no clear active mental illness (such as mania or psychosis) or other conditions that would preclude ability to give informed consent.

Prior to interviews and focus groups, the researchers reviewed the letter of informed consent with participants. Interviews began with the researchers providing a brief presentation about the purpose of the study, the voluntary nature of participation, and the need for mutual respect for individuals and differences of opinion. One of the field researchers acted as a moderator for the group discussions while the other recorded minutes and field notes, including observations of non-verbal communication such as body language and facial expressions [25]. The interviews and focus groups were audio recorded, then transcribed verbatim by a professional transcriptionist.

Data was collected by two researchers (DC and KG). Both researchers had previous experience conducting qualitative interviews and focus groups during graduate training. Both were medical students at the time of data collection and neither had pre-existing relationships with participants, except for the provider informants.

### Study participants

Two focus groups were conducted, one at the DI with their clients (n = 4), and the other at a central location with clients from several organizations (n = 6). Participants in both focus groups were all male, reflecting the preponderance of males in Calgary’s homeless community [1]. One additional interview was conducted with a female client from one of the organizations. This woman also represented the community of homeless families in Calgary as she and her husband had children living with them while they were experiencing homelessness. These individuals were recruited to participate in the study by responding to recruitment posters which were placed strategically in public locations in shelters and homeless service providing agencies.

At the DI, individual interviews (n = 6) were conducted with three clinical staff, two programs staff, and one executive. One participant (physician, nurse or executive) from each of several other agencies (n = 5) was also interviewed individually. These individuals were contacted directly and asked to participate in the study. In total, 22 participants took part in the study (11 clients and 11 providers).

### Data analysis

Data analysis began by establishing a coding template based on relevant literature. Data was coded using these templates; new codes were added inductively as each transcript was coded [26]. Earlier transcripts were then reviewed to check for data that could fall under these new codes. Two researchers independently coded transcripts then met to discuss their initial coding, and to outline themes and patterns that appeared in the data [27, 28].

Member-checking was accomplished by requesting feedback on a four page summary report. Each participant was asked if they wanted to receive such a report. Unfortunately, due to logistical challenges, we were unable to provide this report to the majority of client participants. The feedback we received in response was considered new data, and were incorporated into the final write-up [29].

Trustworthiness was established through several aspects of the methodology, including having multiple researchers code transcripts and compare their findings; member-checking; and the selection of participants through purposeful sampling [30].

### Ethics approval

This research protocol was approved by the Conjoint Health Research Ethics Board at the University of Calgary’s Faculty of Medicine. The researchers adhered to all of its guidelines and policies.

## Results

A summary of our themes and the types of participants stating these themes can be found in Table 1. We present our major findings as per our objectives – firstly, needs for healthcare services among individuals experiencing homelessness in Calgary, and secondly, barriers they face to obtaining necessary healthcare.

**Table 1:** Frequency of themes by participant type

	Providers (/11 interviews)	Clients (/3 interactions^a^)
Needs		
Medical services	10	3
Addictions & mental health	9	3
Allied health services	8	2
Family medicine	7	1
Urgent care	2	0
Chronic disease management	6	0
Social determinants of health	4	3
Barriers		
Patient-level	11	3
Emotional barriers	11	3
Knowledge & priorities	8	1
Provider-level	5	3
Environment & discrimination	4	2
Geographic location	3	2
System-level	11	3
Financial barriers	7	2
Other structural barriers	11	3

^a^2 focus groups & 1 individual interview

### Needs for healthcare services

The majority of providers who participated in this study described the unmet health needs of Calgary’s homeless population as overwhelming. The needs elicited can be grouped into two main categories: medical services; and determinants of health.

#### Medical services

Participants repeatedly mentioned two specific themes related to medical services: addictions and mental health, and allied health services. The other medical services themes that were cited included family medicine, urgent care and chronic disease management.

*Addictions and mental health:* Addictions and mental health were the most commonly cited health concerns of Calgary’s homeless populations as well as some of the greatest needs mentioned. These topics were mentioned in nearly every interview and focus group that was conducted, such as this provider:

*Overwhelming mental health [needs], like multi, multi, multiple diagnosis, all at the same time, that result in behaviors that influence the relationships that the patients have with us. It’s not just mood disorders. There’s a lot of disorder issues that come about from substance abuse and in my population we have a significant population of individuals with mood and substance and thought disorder all at the same time, all overwhelmed, all not coping well in the system.* (male provider 1)

Most informants commented that while they did not know the prevalence figures, they believed that mental illness was highly prevalent amongst homeless individuals: *“number one is the alcohol and drug addictions. Super huge problem that is super hugely neglected or ignored and nobody wants to touch it”* (male client 4). It was suggested that due to the stressful conditions of living on the street, there were high rates of depression and more rapid decompensation from other psychiatric disorders.

The lack of mental health services was believed to act synergistically to reduce overall health outcomes, as explained by one respondent:

*Mental health is a huge part of our client concerns and one of the biggest barriers. It often is a real issue in being able to be effective in helping address their physical health concerns when the mental health concerns aren’t addressed first because you don’t get the same kind of response and cooperation… And that’s a big issue.* (female provider 1)

Some clients expressed concerns that care providers do not adequately assist those with addictions and mental health. Several clients posited that some providers may believe addiction is a self-inflicted problem, and as a result they would be less inclined to provide assistance: *“anybody who goes into any medical facility and has to say the words Drop In Centre, [other shelter name], no fixed address… immediately any source of sympathy seems to slide”* (male client 1).

*Allied health services:* Beyond care from medical doctors, respondents indicated that homeless individuals have a great need for allied health services. These include: nursing, dentistry, optometry, pharmacy, and rehabilitation.

At the time of data collection, the DI had one full-time and one part-time nurse, but the medical staff there wished to have 24-h nursing coverage in the facility. Specifically, they had hoped to initiate a home parenteral therapy program for clients who require intravenous medications or fluids, as at the time such patients had to be sent to a provincial health facility. Wound care is another important nursing contribution. Serious wounds are commonplace; respondents acknowledged their etiology is multifactorial, due to issues like poorly managed diabetes, drug use and poor foot hygiene *“foot problems… street feet”* (male client 4).

The need for dental care was repeatedly mentioned. There were only two organizations that provided dental care for Calgary’s homeless. Several providers mentioned that other medical problems, such as infections and malnutrition, may result from poor oral health due to a lack of available dental care. One provider lamented that extraction is often the only definitive treatment offered. The reason for this was explained: *“Because it is so complicated to take care of a tooth, the patient has to come in on a regular basis and you need to know that you can follow up with this patient… and that can be a problem”* (female provider 2).

Alberta’s public health insurance does not cover optometry or corrective lenses, making the costs associated with these prohibitive for most individuals experiencing homelessness: *“Lots of people have simple myopia like me, and they just need a pair of glasses, but that’s not so easy if you don’t have any money or any insurance”* (female provider 2).

Beyond managing medications, many clients noted that prescription coverage is not available through the Alberta Health Care Insurance Plan (ACHIP), and most patients pay out of pocket for their medications. There is provincial funding which will pay for emergency prescription coverage for up to three months, however this is insufficient for those suffering from chronic mental and physical ailments: *“another problem I’ve found is for your medication… social services will only pay a few times and then you’re not covered anymore… and I kind of ran out”* (male client 7). While some people qualify for ongoing healthcare support, applying for this can be complex, and having social workers to assist with the process would be helpful but is not universally available.

#### Social determinants of health

Many stakeholders specifically recognized that the social determinants of health were at the root of the illnesses and afflictions commonly seen in homeless populations:

*I think that patients are much more complex than they are in a typical family practice… you’re not just addressing one of the determinants of health. I think you want to look at all of the determinants of health and see if you can make an impact on any of those as well.* (female provider 2)

Some of the social and environmental factors that were mentioned were: income and income support; nutrition and food security; and housing and environmental concerns in shelters.

Income is often regarded as the most important determinant of health [31]. For most individuals experiencing homelessness, including the working poor, their income is insufficient to satisfy their needs for housing, food and other basic necessities. Many homeless Albertans could be eligible to receive income support. Unfortunately, many do not know how to apply for these programs:

*The other thing that they come to us for are issues around income, income security. So that often while they’re adults, [they’re] not necessarily eligible for significant income from Social Services. So we spend a lot of time to, to sort out what is their best access to, to a stable income.* (male provider 1)

Several participants spoke about how food insecurity can lead to malnutrition and worsening of health status. Clients who have lived in homeless shelters lamented the difficulty they experience in trying to eat a nutritionally well-balanced diet. They reported that while shelters often do provide adequate caloric intake, they perceive that the meals are not particularly healthy. It is also difficult to access nutrition information: *“one of the things I notice about the [shelter] meals is that it’s basically there is no nutritional information. So I have no idea how much salt. I can’t find out how much salt I’m getting per day, how many calories I’m getting, that sort of thing.”* (male client 7).

One environmental factor that was mentioned was recycling of air and the possible transmission of communicable diseases: *“Now it has to do with the air conditioning being recycled directly into the building. If you were at [shelter name] two years ago, if you were in that place more than two weeks, you start to cough. It was, it was a wonderful little lung infection that stayed with you until you were at least three to four weeks outside of [the shelter]”* (male client 1).

Another environmental factor described was a lack of safe needle deposit boxes: *“they don’t have the dispensers for needles, especially at the shelters. I see them laying around you know. The drop boxes… they don’t have them”* (male client 5).

### Barriers preventing access to healthcare services

Both stakeholder groups interviewed in this study identified the existence of barriers. These can be characterised into three groups: patient- , provider- , and system- level.

#### Patient-level: emotional barriers

It has been reported that fear of bad news presents a general barrier to accessing care [32]. Due to the complex life stressors faced by individuals experiencing homelessness, this may prevent some from seeking care. This fear is further complicated by the fact that many homeless people do not have a support system upon which they can draw if they receive a poor prognosis or alarming diagnosis:

*They’re already in a stressful situation in their life being homeless. The stress level is incredible so to throw in a health issue would just increase that stress more than they can possibly bear without support. They might be scared to go to the doctor in case something that they can’t handle arises. Lack of social support could also mean they feel like they have no one to lean on if they do get bad news…* (female provider 3)

Another negative emotional experience clients may have is fear of their provider. The clinical relationship is one with an inherent power dynamic, and feeling subordinate in that relationship can arouse feelings of fear in patients. One provider elaborated:

*Most of our people have a fear of authority. Medical systems are structured to represent that. They're incredibly hierarchical and even physically they're set up to be daunting to get through… a lot of our clients will hide ailments and I think that just comes from a lifetime of fear of authority.* (female provider 1)

Feelings of shame, low self-esteem and worthlessness are prevalent amongst individuals experiencing homelessness [33]. Clients acknowledged the apprehension that their peers experience disclosing to healthcare professionals that they are homeless:

*The psychological barrier of having to walk in and say that you're homeless. Part of it is our fault, part of it is us turning around and feeling uncomfortable and projecting that when it happens. The other half is a definite, darker side of the medical community that turns around and goes ‘oh, is that what you are?’* (male client 1)

#### Patient level: patient knowledge and priority setting

As many informants noted, preventive healthcare is often deprioritized in favour of managing other more acute issues:

*I think one of the biggest gaps is looking at that point of access as to what really is their need and not placing on them that we think they need. Today it might be only about Joe's meal. He really doesn't care that his immunizations are not up to date.* (female provider 4)

Informants noted that lack of education about prevalent illnesses among the homeless community also created a barrier in accessing care, in that individuals are often unable to identify that a problem even exists and are therefore not motivated to seek care. One client commented: *“there seems to be a lack of education in a lot of the clients, or people who are homeless and somehow we’ve got to get across to them exactly how to reduce the spread of viruses such as colds and flus and other kinds of ailments that involve the transference of bacteria”* (Male client 3). This participant went on to say: *“You, you need, you need to give them information, try to break it down to their level”* (male client 3).

#### Provider-level: environmental barriers and discrimination

Informants identified that there are a multitude of environmental barriers in existence that limit accessibility. Some of these barriers include the location of medical services, and the atmosphere within clinics or hospitals. Individuals may be less willing to go to a clinic if it is located in an area they are unfamiliar with or feel uncomfortable in because of the potential for greater public and/or police surveillance and control. A provider described this in the context of one clinic:

*A lot of times they’ll go down to [clinic] and if they’re not waiting eight hours, they feel very outcast there. They don’t feel included. Staff can be very rude or judgmental to them there. I mean we had [another facility], they felt more comfortable there because it wasn’t as out of their comfort zone I guess you could say, as opposed to like you know when they go down to those nice brand new buildings.* (female provider 7)

Thus, this facility in a ‘nice brand new building’ was frequently described as being less welcoming to the homeless community because of features such as a reception check-in and security guards in the waiting room. One provider claimed that she had heard stories of patients seeking medical care who were *“hassled by security and they get kicked out right away before they can get assessed”* (Male provider 2). Regarding the reception desk and check-in procedures, one provider stated: *“It is not a physical barrier to you and I, but to the marginalized and homeless, that is a physical barrier and they will sooner walk away than face that barrier so less people go to [the clinic]”* (female provider 5)

Informants expressed that clients felt they had received poor care, including a lack of understanding on the part of healthcare providers of their social context and ongoing stressors in their lives:

*Discrimination [is a big problem]… If you were living in a shelter, no fixed address, then you’re poor, you work sex trade, you’re using drugs, you’re HIV positive, many, many things, you’re discriminated against even by healthcare.* (female provider 6)

One client participant recounted a story of what he felt was discrimination by an emergency physician:

*I ended up at the [urgent care center], yeah, I went there and then they says no, you’re really in trouble, your lungs. So they admitted to the [hospital] and the doctor there said ‘I’m admitting you’, he had all the papers. He comes back an hour later and said ‘what’s your address?’ I said the [shelter]. Then he says ‘well just a minute…’ He went and comes back and said ‘I, I looked at your vitals and you’ve come up such a long way since you were diagnosed five hours ago, that I’m going to let you go,’ at three in the morning… because I said I was staying at the [shelter], I’m pretty sure.* (male client 6)

Healthcare providers may also make inaccurate assumptions about how the living conditions of their homeless patients affects their ability to maintain their health. One client stated: *“When you tell them that you’re homeless, I think they find that it’s falling on deaf ears because why would they give you the health awareness when you live in this environment that’s filthy as it is”* (Male client 2). Issues such as a lack of transportation or money can be overlooked by healthcare providers when patients are discharged home, leading to a negative emotional experience and reluctance to return to care in future. One client told of how a peer was discharged from hospital in the middle of the night with no means of transportation or supports to call upon, and was forced to walk home.

Negative past experiences with healthcare providers were often cited as contributing to a sense of mistrust and reluctance to disclose personal information to health professionals. One provider stated: *“We don’t have a great sense of understanding about this population or their needs and so they either get under served or inappropriately served”* (female provider 1) another suggested that we need to *“educate the [providers] within the medical system to be more sensitive to folks who are coming [from] a chronic homeless situation”* (male provider 3).

We were told that even if an individual has not had a poor healthcare experience, others’ experiences may be transmitted via word of mouth and lead to apprehension and fear of healthcare centres in general. Clients recounted stories they heard of individuals being kicked out of healthcare facilities, or being treated poorly by providers. One example of such a second hand account of a negative experience: *“I heard about an individual who was at [urgent care] getting help with some of kind problem that he has that involves a lot of pain. The moment they heard he was from a shelter, all they prescribed for him was Tylenol. They avoided the ones with codeine because they figured ‘oh, either he or somebody else in the shelter will abuse it.’ The moment you go and mention it, bang! Somehow the treatment level gets discounted down”* (male client 3).

In addition to the stigma associated with being homeless, members of other sub-communities are also subject to discrimination that can negatively impact their care. One provider singled out the Aboriginal homeless community as one that faces significant discrimination. Informants in this study made specific reference to the Aboriginal homeless community and how members are subject to discrimination on the basis of their ethnic background:

*Attitude of staff is a barrier. The attitudinal issues that a staff has regarding the nature of who that patient is – that’s racism, bias, all that kind of stuff. Discrimination exists for the homeless population in general and the Aboriginal homeless population. I think that's a primary issue that generates how an individual accesses a system or turns away from a system after they've accessed it. The perception that they're not going to be treated well is part of a series of access barriers.* (male provider 1).

#### System level barriers: financial barriers

Some commonly cited financial barriers deterring homeless individuals from accessing healthcare include: money for transportation, health benefits, or coverage for prescriptions and allied health services.

Individuals experiencing homelessness are less able to afford transportation to clinics or hospitals, which is especially problematic in a city as large as Calgary. There are currently few resources available to assist individuals with transportation to and from clinics that are not located in easily accessible locations:

*Patients get here but then they request a ticket to go home either a taxi or a bus, so we struggle with our responsibilities for that. It shouldn't necessarily [be] a policy that should apply to everyone, it needs to be based on where they live, what the physical disability is, all that kind of stuff.* (male provider 1)

The AHCIP covers inpatient hospital costs and physician visits. Unfortunately some homeless individuals do not have AHCIP coverage, or are unsure if their insurance from other provinces is transferrable to Alberta: *“I was concerned because I don’t have like, ah, what’s you call? Healthcare up here ‘cause I still have one in Quebec so… all the legal stuff… so I was kind of worried about that when I went to the hospital”* (male client 8).

Beyond the basic costs covered by AHCIP, Albertans are responsible to pay for other healthcare costs, including medications. Most homeless individuals do not have an extended insurance plan to access allied health services or purchase pharmaceuticals. Informants acknowledged that even if homeless individuals would be eligible for some public benefits they wouldn’t know how to go about accessing these: *“right now I got Alberta Health but, I’m trying to get, Alberta Seniors [benefit]. Now they won’t, ah, give it to me now. I don’t know why. That’s what’s holding me back. I had to go through a whole bunch of paperwork to get it I guess. I don’t know what now”* (male client 9).

#### System level barriers: other structural barriers

These barriers largely relate to health system organization and include: patients’ lack of identification, scarcity of resources leading to inconvenient clinic hours; and navigation.

Many stakeholders, including both providers and clients, described that an important barrier to accessing healthcare services is a lack of government-issued identification, including their provincial healthcare card: *“One of the things I just thought of that could be a potential barrier is missing or stolen ID”* (male client 1). One provider stated:

*Identification is something that you often need when you go to clinics and a lot of our [clients] do not have ID - whether or not they even have Alberta Health Care cards with them or have even applied for their Alberta Health Care cards. We have a lot of out-of-province [clients] that come through, a lot of immigrants that come through so then that whole issue is do they even get access to certain types of care just due to not having the proper documents.* (female provider 8)

Clinic hours repeatedly arose as a key structural barrier. Many members of Calgary’s homeless population are considered “working poor” and cannot afford to take time off of work to visit clinics which are only open during regular business hours. One provider stated:

*[One organization] is only running from seven to three so if you're working during those hours you're out of luck. [Another provider] is fully open 24 hours a day but [they] triage according to urgent care levels so they may be waiting all night for a simple question.* (female provider 3)

Navigation through the complex healthcare system presents a substantial barrier, particularly if patients are unable or unaware of how to advocate for themselves to receive the care they require, as expressed by one of our provider informants:

*They're not terribly good self-advocates, and may not have the literacy or educational background to be able to navigate or interpret the medical system in a way that serves them well, so if someone tells them no at an entry point to a clinic or any other medical service, they just tend to back away and go rather than ask the question about whether there's another option or whether they've [been] misunderstood.* (female provider 1)

## Discussion

Respondents identified currently unmet needs related to medical services, as well as addictions and mental health. It was suggested that there is an unmet need for more service provision from allied health professionals, and that these services should be provided in such a way as to be sensitive to the social determinants of health that have contributed to and perpetuated the difficult living situations of those experiencing homelessness.

Our findings add to the sparse qualitative literature on the health needs of individuals experiencing homelessness in Canadian cities. These findings add depth to previously conducted quantitative studies [8–11, 34, 35]. Our study’s findings around the healthcare needs of homeless populations are largely consistent with those previously conducted in the UK [12–14] and USA [15–17]. However, many of the barriers to accessing care that we found in our study were uniquely Canadian. For example, participants described given that they experienced financial barriers to accessing medications and allied health services, which is a direct result of Canada’s publicly funded health insurance system which covers hospital and physician expenses, but not outpatient medications or allied healthcare providers’ fees.

One important finding was the suggestion that an effective provider must also act as an advocate on behalf of their patients, exemplified by one respondent’s comment that *“You need to be up to speed on everything that the population comes through the door with, but you also need to know how to effectively be an advocate”* (male provider 1). Furthermore, solutions that successfully address the social determinants of health will require looking outside the traditional scope of practice of the healthcare system, and will likely include addressing income and social support, as well as housing.

While physician care for chronic disease is important, more complex interventions including team-based approaches to disease management have been shown to yield superior outcomes [36]. These programs may involve team members such as registered nurses with specific training in chronic disease management, and patient education. Although these services have recently started to be provided in Calgary, through the DI as well as other service providers with funding support from Primary Care Networks (an Alberta primary care initiative to improve family physicians’ capability to provide allied health services) [37], respondents indicated there are still unmet needs in relation to mental health and chronic disease management. Inter-professional models of care provision, such as the patient-centred care home [38] have been shown to reduce costs and improve outcomes [39], and may be beneficial for this population.

We identified barriers to meeting these aforementioned needs, at patient, provider, and systems levels. Emotional barriers- the fear or apprehension of receiving bad news- were suggested to play a significant role in the decision to not access care. This interacts with the other identified personal barriers: priority setting and patient education. It was suggested that homeless individuals may not have ideal priority setting for whether to access health services, and that a lack of education about what symptoms or situations constitute a valid reason for presenting to services, may contribute to poorer health status. This use of health information to make decisions about accessing health services is known as “health literacy” [40] and it is well-established that individuals with lower health literacy have poorer health outcomes [41]. Provider-level barriers were identified including environmental barriers and discrimination. If the environment around and inside the clinic is not welcoming, or discriminatory towards the homeless community, people will not feel comfortable accessing the care they need. This is even more important for those who have had previous negative experiences with a health service. One way of mitigating this may be to provide services in locations that are already trusted by the homeless community. Finally, system issues affect individuals’ ability to access healthcare services, particularly issues around financial barriers to transportation as well as medications and allied health services; obtaining government issued identification; and navigation of the complex healthcare system.

This study has limitations that have implications for the trustworthiness and transferability of the findings [29]. The study was located in only one city and the results may not be transferrable to other locations with different health systems. However, Calgary is a relatively affluent and well-serviced city with a nationally respected plan and lead organization, the CHF, and it is unlikely that the homeless are dramatically better served in other cities. Furthermore, we collected data from both service providers and from clients – therefore, the perceived needs of service providers may not be entirely congruent with those actually perceived by the service users. However, in Table 1 we demonstrate that our main themes were raised by both providers and clients. The problem of ascertaining when data saturation is reached is one that continues to be debated widely. Broadly, it is acknowledged that it is a matter of judgment [42] and that either informational redundancy [29] or theoretical saturation [43] is the goal. Given the challenge in recruiting participants from several organizations as well as homeless individuals, recruitment did not proceed iteratively; rather, all participants were identified a priori. Although some authors have suggested a specific minimum sample size of 30 is required for this type of inquiry [44], others have noted that an appropriate sample size is that which adequately addresses the research question [43]. During the last few interviews, few new themes emerged, so it is likely that data saturation was attained. While we felt that our data had reached saturation it is possible that a larger sample may have yielded more themes. In addition, this study represents a snap-shot in time of access to primary care amongst homeless populations, and the situation could worsen (or improve) with sudden changes in provision of housing or primary care services. Finally, by design, this study did not evaluate the health status of Calgary’s homeless populations.

## Conclusion

We have identified needs and barriers to accessing primary care for Calgary’s homeless population. Despite the fact that Canada’s healthcare system is publicly funded and purportedly equitably distributed, there remain significant and important unmet needs for these particular populations.

In summary, the rich data that came from discussions with informants illustrated that the barriers to accessing care operate on multiple levels, and are significant enough that they very likely impact health outcomes and well-being. Barriers at all levels must be addressed if primary care among the homeless is to be improved. Innovative solutions are urgently required that transcend the traditional silos of medicine, nursing and social work, and that build on the understanding of a variety of stakeholders, including service users themselves. We suggest that by taking the time to ensure that homeless individuals have direct input into the development of services, their unmet needs will be addressed and barriers to accessing essential services will be more effectively reduced.

## Abbreviations

AHCIP: Alberta Health Care Insurance Plan
CHF: Calgary Homeless Foundation
The DI: Calgary Drop In and Rehabilitation Centre
